# FT-IR Microspectrometry Reveals the Variation of Membrane Polarizability due to Epigenomic Effect on Epithelial Ovarian Cancer

**DOI:** 10.3390/ijms151017963

**Published:** 2014-10-08

**Authors:** Morris M. H. Hsu, Pei-Yu Huang, Yao-Chang Lee, Yuang-Chuen Fang, Michael W. Y. Chan, Cheng-I Lee

**Affiliations:** 1 Department of Life Science, National Chung Cheng University, Min-Hsiung, Chia-Yi 62102, Taiwan; E-Mails: morris679@gmail.com (M.M.H.H.); cindy0120811@yahoo.com.tw (Y.-C.F.); biowyc@ccu.edu.tw (M.W.Y.C.); 2 National Synchrotron Radiation Research Center, Hsinchu 30076, Taiwan; E-Mails: pyhuang@nsrrc.org.tw (P.-Y.H.); yclee@nsrrc.org.tw (Y.-C.L.)

**Keywords:** FT-IR, methylation, paraffin-adsorption, ovarian cell, 5-aza

## Abstract

Ovarian cancer, as well as other cancers, is primarily caused by methylation at cytosines in CpG islands, but the current marker for ovarian cancer is low in sensitivity and failed in early-stage detection. Fourier transform infrared (FT-IR) spectroscopy is powerful in analysis of functional groups within molecules, and infrared microscopy illustrates the location of specific groups within single cells. In this study, we applied HPLC and FT-IR microspectrometry to study normal epithelial ovarian cell line immortalized ovarian surface epithelium (IOSE), two epithelial ovarian cell lines (A2780 and CP70) with distinct properties, and the effect of a cancer drug 5-aza-2'-deoxycytidine (5-aza) without labeling. Our results reveal that inhibition of methylation on cytosine with 5-aza initiates the protein expression. Furthermore, paraffin-adsorption kinetic study allows us to distinguish hypermethylated and hypomethyated cells, and this assay can be a potential diagnosis method for cancer screening.

## 1. Introduction

Epithelial ovarian cancer causes more deaths than any other cancers in the female reproductive organ in the United states and Europe [1]. Because of the lack of early detection strategies, many ovarian cancer patients present with advanced stage disease, and the overall 5-year survival for these women is only 30% [2]. Current diagnosis of ovarian cancer relies on serum marker CA-125 which is also known as MUC16, a transmembrane protein encoded by the *MUC16* gene [3,4]. The CA125 is elevated in other cancers including endometrial, pancreatic, lung, breast, and colon cancer, and in menstruation, pregnancy, endometriosis, and other gynecologic and non-gynecologic conditions [5]. Moreover, CA-125 lacks the sensitivity to detect early stage cancers. Because of the low prevalence between ovarian cancer and the current marker, more sensitive and specific diagnosis methods are required.

**Figure 1:**
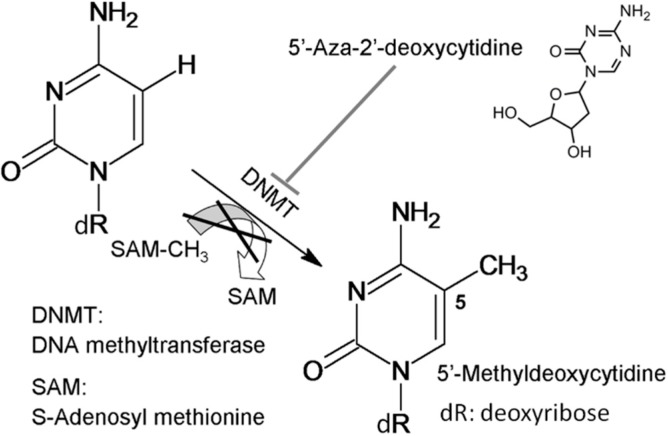
Reaction scheme of DNA methylation.

The gene expression is primarily determined by the inherent genome sequence, but can be affected by non-inherent DNA methylation known as epigenetic modification. In the mammalian genome, methylation occurs only at 5' position of cytosine bases in a CpG (cytosine and guanine separated by a phosphate) dinucleotide [6]. Methylation of CpG islands can be classified into hypomethylation and hypermethylation. Cytosine methylation is carried out with the assistance of DNA methyl transferases (DNMTs) and with methyl–donation from *S*-adenosyl methionine (SAM) as shown in Figure 1. Methylation of CpG inhibits the activities of transcription factors, and therefore inhibits the gene expression. In the current cancer therapies, 5-aza-2'-deoxycytidine (5-aza, trade name Dacogen, a cytosine nucleoside analog) is common used to inhibit DNA methylation [7].

To develop a diagnosis method for screening ovarian cancer, normal and ovarian cancer cell lines with distinct methylation level were used. As most human ovarian carcinomas were demonstrated to be experimentally risen in the ovarian surface epithelium (OSE) [8,9], an immortalized OSE (IOSE) cell line was generated from normal OSE by transfecting simian virus 40 large T antigen. The immortalized ovarian surface epithelium (IOSE) lines resemble cells which are involved in early neoplastic progression, and they should be useful to study ovarian carcinogenesis [10]. The A2780 human ovarian cancer cell line was established from tumor tissue originated from an untreated patient. Compared with IOSE, the overall 5-methylcytosine content of the genome and methylation at satellite DNA sequences in A2780 cell line decreased frequently. Specifically, at many CpG islands, which overlapped promoters of tumor suppressor genes, the methylation hierarchy of A2780 was higher than IOSE [10,11,12,13,14]. Cell line CP70 derived from parental cell line A2780 was eight-fold more resistant to cisplatin. During DNA synthesis the newly synthesized daughter strand will include errors commonly. Mismatch repair (MMR) is a system responsible to recognize and repair incorrect insertion, deletion and misincorporation of bases that can arise during DNA replication and recombination. Cell line A2780 is MMR proficient and expresses mismatch repair protein MLH1, whereas CP70 is MMR deficient and does not express MLH1 protein because of hypermethylation of the *hMLH1* gene promoter [15]. Loss of MMR due to methylation of the *hMLH1* gene promoter results in resistance to cisplatin in cell lines *in vitro* and in human tumor xenografts *in vivo* [16]. Overall, cell lines IOSE, A2780 and CP70 are representative in the studies of ovarian carcinogenesis.

Proteins associated with cancer cell plasma membranes play key roles in the abnormal signal transduction processes required for carcinogenesis. Cancer membrane-associated proteins have been targeted to develop cancer therapeutics, such as herceptin (her2neu) [17], Panorex (Ep-CAM) [18] and IRESSA (epidermal growth factor receptor) [19]. Therefore, cell membranes are perfect target for us to develop specific diagnosis methods for ovarian cancer.

**Figure 2:**
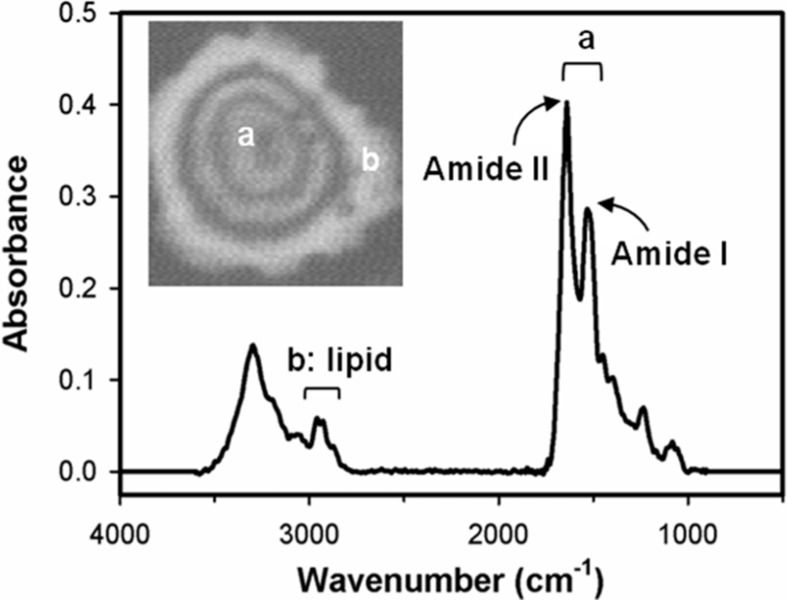
The chemical information of biomolecular distribution within a cell including amide I (~1540 cm^−1^) and amide II (~1640 cm^−1^) of proteins detected in nucleus and lipid (2800–3000 cm^−1^) shown by fourier transform infrared (FT-IR) spectrum and microscopy image (inserted figure).

Infrared (IR) spectroscopy is a powerful tool to analyze the functional groups within molecules based on the distinct energy of each vibration mode. When Fourier transform infrared spectroscopy (FT-IR) is applied to microorganisms or tissue sections, the chemistry of small areas or even single cells can be detected by spatially resolved infrared microspectrometry, the combination of FT-IR spectroscopy and microscopy. As shown in Figure 2, the IR-absorption of amide I (C=O stretching, 1520–1560 cm^−1^) and amide II (-R'-NH stretching, 1630–1690 cm^−1^) rising from protein backbones is rich in nucleus of the cell, whereas the IR-absorption of lipid (2800–3000 cm^−1^) is rich in the cell membrane. In contrast to most conventional detection methods, FT-IR microspectrometry does not require additional reagents or stains and can be performed without tissue homogenization or chemical modifications on measured samples. Therefore, FT-IR microspectroscopy is a rapid, direct, and non-destructive analytical technique to study molecular chemical features of biological samples. To enhance the spatial resolution of biochemical events associated with disease progression, synchrotron-based FT-IR microspectrometry has been applied to differentiate various types of cancers [20,21,22] and probing the malignancy development and progression [23,24,25]. Taking the advantage of excellent signal-to-noise ratios, synchrotron-based FT-IR microspectrometry has been widely utilized to study the biochemical components in biomedical applications, such as the relative lipid and protein content of the cells during the cell cycle [26]. Based on the differentiation of the lipid components of the cells, an innovative methods of wax-adsorption infrared kinetics was developed to differentiate normal cells from premalignant and cancerous oral epithelial cells based on the vibrational signals of CH_2_ and CH_3_ [27].

In this work, we conducted high-performance liquid chromatography (HPLC) and FT-IR microspectrometry to analyze the effect of 5-aza on DNA methylation and protein expression in three representative cell lines. To furthermore distinguish normal and ovarian cancer cells, we applied wax-adsorption kinetic study with the use of paraffin and beeswax as a preliminary development of ovarian cancer detection.

## 2. Results and Discussion

### 2.1. 5-Aza-2'-deoxycytidine (5-aza) Weakens Methylation of Deoxycytidine Monophosphate (dCMP) and Enhances Protein Expression in CP70

**Figure 3:**
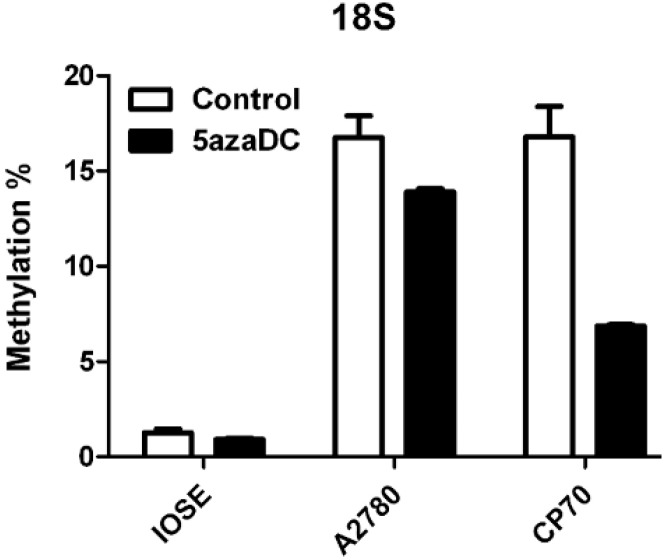
Treatment of ovarian cancer cell lines with 5-aza-2'-deoxycytidine (5-aza). The methylation level of ribosomal DNA (rDNA) in 18S was determined by real-time reverse transcription PCR.

Deoxycytidine monophosphate (dCMP) and methylated dCMP are the critical epigenomic components in cancer cells. Methods for DNA methylation analysis can be divided roughly into global and gene-specific methylation analysis. For global methylation analysis, the overall level of methyl cytosines in genome is measured. Chromatographic methods and methyl accepting capacity assay are examples of global-methylation analysis. For gene-specific methylation analysis, a large number of techniques have been developed, while the bisulfite reaction based methods have become very popular such as methylation specific PCR, bisulfite genomic sequencing PCR. A further biological approach of global methylation analysis has been developed by detection of ribosomal DNA (rDNA) methylation [28]. Based on the detection of 18S rDNA as shown in Figure 3, DNA methylation level in A2780 and CP70 is significantly higher than IOSE, and the level of methylation can be weakened by 5-aza treatment, especially in the case the CP70 that more than half of methylation is inhibited. Hypermethylation of ovarian cancer cells observed in this study is different from hypomethylation reported previously [29]. This difference could be due to the variation of the cell lines.

In addition to the analysis of 18S rDNA, we have tried quantitative analysis of DNA methylation by HPLC detection of dCMP and methylated dCMP (mdCMP). The detection limit of this method can reach 1 µg of DNA [30] and the chromatogram only takes few minutes. In this HPLC analysis, the DNA methylation is detected upon the whole genome, while the specific gene sequence is targeted in the bisulfite assays. As shown in Table 1, the retention time for dCMP and methylated dCMP is 3.6 ± 0.1 and 4.0 ± 0.1 min, respectively. The ratio of methylation is low at 4.3% in IOSE, and treatment with 5-aza has no significant effect on methylation as predicted. In contrast, in A2780, the methylation level is 5.4%, and 5-aza treatment decreased 76% of the methylation ratio to 1.3%. The ratio of DNA methylation in CP70 is higher (7.7%) in comparison to the ratio in IOSE and A2780. Treatment with 5-aza significantly decreased ~38% of the DNA methylation. The values of DNA methylation ratio in HPLC detection are lower than the values measured in quantitative real-time methylation specific PCR (qMSP) of 18S rDNA, but the trend of 5-aza effects is consistent in two methods. Different from the specific gene sequencing, in this analysis of the whole genome, 5-aza is more effective in A2780 than in CP70.

**Table 1:** Effect of 5-aza by high-performance liquid chromatography (HPLC) quantitation on deoxycytidine monophosphate (dCMP) and methylated dCMP (mdCMP) in immortalized ovarian surface epithelium (IOSE), A2780 and CP70 cell lines.

Sample	Treatment	Retention Time (min)		Ratio of Methylation *^a^*
		dCMP	mdCMP	
Standard		3.6 ± 0.1	3.9 ± 0.1	
IOSE	None	3.6 ± 0.1	4.1 ± 0.1	4.3% ± 1.7
	5-Aza	3.6 ± 0.1	3.9 ± 0.1	4.2% ± 0.8
A2780	None	3.5 ± 0.1	4.0 ± 0.1	5.4% ± 2.4
	5-Aza	3.6 ± 0.1	4.1 ± 0.1	1.3% ± 0.1
CP70	None	3.6 ± 0.1	4.0 ± 0.1	7.7% ± 2.0
	5-Aza	3.6 ± 0.1	4.1 ± 0.1	4.8% ± 0.6

*^a^* Ratio of methylation was determined as follows. A_mdCMP_/(A_dCMP_ + A_mdCMP_), A = integration of the absorption at 254 nm. The reported values were calculated from three independent measurements.

The effect of 5-aza was also observed in protein expression based on the integration of IR signal within fixed area between 1470 and 1730 cm^−1^ containing stretching of amide I and amide II rising from protein backbones, and the signal collected from 20 single cells in each type of cell was summarized in Table 2. In IOSE, the level of protein expression increased slightly (~10%) after 5-aza treatment. This is correlated with the small decrease (~10%) of methylation upon 5-aza treatment. Similar to the demethylation effect in Figure 3, this 5-aza treatment significantly increased the protein level for ~30% in A2780 and ~50% in CP70. Notably, the calculated protein level includes proteins on the membranes and in nuclei of the cells. Consistent with the methylation level detected in the specific gene sequence (Figure 3), the effect of 5-aza is the most pronounced in CP70. The degree of 5-aza effect would not be exactly the same in protein and methylation levels. The data of DNA methylation and protein level are inversely correlated in three cell lines. The function of 5-aza is known to induce hypomethylation of DNA. Taking together the known function of 5-aza and the result of protein level, it is predictable that demethylation on CpG island of promoter induced gene expression and consequent production of proteins [31].

**Table 2:** The level of protein expression in cell lines IOSE, A2780 and CP70 determined by integration of IR signals of amide I and amide II.

Cell Line	No Treatment	5-Aza-Treatment
IOSE	13.7 ± 1.2	15.1 ± 2.1
A2780	11.9 ± 1.0	15.6 ± 3.4
CP70	11.3 ± 1.4	17.8 ± 2.0

### 2.2. Distinguishing Ovarian Normal and Cancer Cells with Paraffin-Adsorption Kinetics

**Figure 4:**
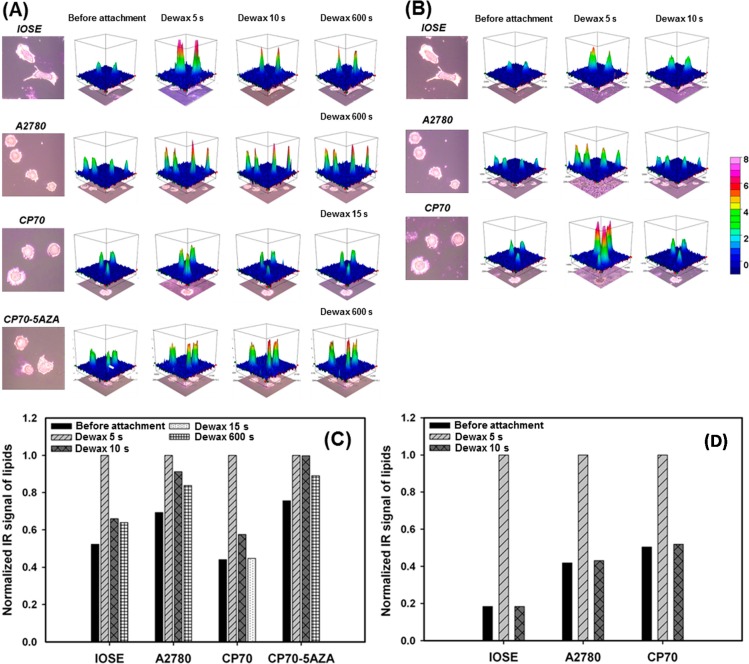
Wax-adsorption kinetic study of (**A**) paraffin (**B**) beeswax compared among IOSE, CP70 and A2780 cell lines at different de-waxing time. De-waxing was performed with xylene-wash after wax-fixing. The amount of wax was determined by integration of absorbance from 2800 to 3000 cm^−1^; The quantitation of (**C**) paraffin or (**D**) beeswax-attachment signal on each ovarian cell line determined by the IR-absorbance from 2800 to 3000 cm^−1^.

After the determination of the overall protein level, we are further interested in the cell membrane because cell signaling is highly relied on the membrane receptors. Many of the membrane proteins are involved in the prognosis of the most common forms of cancer and are the hallmark of a cancer cell [32]. As the cell membranes play key roles in carcinogenesis, we have been aimed on distinguishing the ovarian normal and cancer cells by the variation that might present in the cell membranes between the cell lines. As the distinct features of cell, which consist of lipid bilayers, are hydrophilic and lipophilic, we tested the adsorption of two kinds of wax (paraffin and beeswax) to the ovarian cell lines. The polarizability of paraffin is extremely low for the long symmetrical chain, while the main component of beeswax has unsymmetrical carbon-chains resulting higher polarizability than that in paraffin. From the FT-IR spectra of wax-adsorbed cells, we integrated the IR signal representing lipids (2800–3000 cm^−1^) within each single cell (Figure 2). In addition, the effect of 5-aza was tested in CP70 for paraffin-adsorption, because the protein level of CP70 significantly increased after 5-aza treatment, as shown in Table 2. The IR signal of lipids was recorded when the cells were attached with wax and when the wax-attached cells were dewaxed with xylene (read Section 3.5 for the detail). The dewax was performed at a different duration depending on the properties of the wax-attached cells. In Figure 4A,B, 3D plots illustrate the normalized integration results of cells before wax-attachment and with various dewaxing time. The infrared signal of lipids before wax-attachment indicates the lipid bilayers on the cell membranes, while the lipid signals after wax-attachment represents not only lipid bilayers but also studied wax (paraffin or beeswax). Therefore, the difference between the two conditions indicates the studied wax attached on the cell membranes. The quantitative results were compared in Figure 4C,D.

In the work of paraffin-adsorption (Figure 4A,C), all tested cells had pronounced paraffin-attachment so that significant amount of paraffin was observed after 5 s of xylene-wash. The further wash removed ~40% paraffin from IOSE. Differently, in hypomethylated A2780, attached paraffin on A2780 membrane could not be easily removed by xylene-wash. The similar paraffin-adsorption pattern of IOSE was observed in hypermethylated CP70, but CP70 performed even weaker adsorption that paraffin got washed away completely in 15 s. Once CP70 was treated with 5-aza, the DNA methylation was inhibited, and the paraffin-adsorption became extremely strong. After xylene-wash for 600 s, ~90% of paraffin remained attached to the cell membranes and the adsorption kinetics resembled the pattern in A2780. Overall, the higher the DNA methylation, the weaker paraffin-adsorption was observed. The paraffin-adsorption assay revealed that paraffin-adsorption in CP70 was turned into similar adsorption pattern in A2780 by 5-aza treatment. This result indicates that 5-aza changes the environment of the membrane receptors, the critical part of carcinogenesis.

## 3. Experimental Section

### 3.1. Cell Culture and Drug Treatments

Ovarian cell line IOSE, cancer cell lines CP70 and A2780 were cultured as previously described [33]. After the overnight incubation, cells were cultivated with fresh medium treated with 5 µM of 5-aza (Decitabine, Sigma, St. Louis, MO, USA) and the medium was changed every 24 h until cell harvesting after seven days. For the measurements with FT-IR microspectrometry, the cells were cultivated on MirrIR low-e microscope slides (Kevley Technologies, Chesterland, OH, USA) with adding sodium butyrate to final concentration of 5 μM to keep samples resting in the G1 phase of the cell cycle. Finally, the ovarian cells were fixed on MirrIR low-e microscope slides with 4% formaldehyde.

### 3.2. Quantitative Real-Time Methylation Specific PCR (qMSP)

Bisulfite converted DNA was subject to real time qMSP using ABI StepOne real time PCR system (Applied Biosystems, Foster City, CA, USA) as previously described [28] with slight modification. In brief, each reaction contained 12.5 μL of 2× SYBR green PCR mix (Toyobo, Osaka, Japan), 160 nM of each primers and 4 μL of bisulfite modified DNA in a total volume of 25 μL at 95 °C for 10 min, 40 cycles of 95 °C for 15 s, 67 °C for 30 s and 72 °C for 30 s. Primers targeting CpG island of 18S rDNA and *β-actin* (ACTB) for normalization of input DNA was used as previously described [28]. The amount of methylated 18S and ACTB were determined by the threshold cycle number (*C*_t_) for each sample against a standard curve generated by SssI-treated DNA-MSP cloned fragment. The percentage of 18S methylation was calculated as the 18S: The ACTB ratio of a sample divided by the same ratio of SssI-treated sperm DNA (Millipore, Billerica, MA, USA) and multiplied by 100.

### 3.3. DNA Analysis with Reverse-Phase HPLC Measurement

The DNA samples were extracted from cultured cell lines with tissue and cell genomic DNA purification kit (Genemark Technology, Taichung, Taiwan). The extracted DNA samples (5 μg) were prepared for HPLC analysis based on the procedure developed by Ramsahove [30]. The HPLC analysis was performed with a reversed phase column (250 × 4.6 mm, 5 μm Vydac^®^ 218TP, C18, Grace, Columbia, MD, USA) running through 50 mM ammonium phosphate (pH 4.1) at flow rate 1 mL/min. To confirm the assignment of dCMP and methylated dCMP (mdCMP), commercial dCMP and mdCMP standards (Sigma) were applied.

### 3.4. FT-IR Spectromicroscopy of Ovarian Cells

The FT-IR spectromicroscopy of single cells was captured by Nicolet Magna 860 spectrometer (Thermo-Nicolet Instruments, Madison, WI, USA) and Spectra Tech Continµm IR microscope (Spectra Tech Inc., Oak Ridge, TN, USA) with 4 cm^−1^ of spectral resolution and 900–3600 cm^−1^ of scanning range at National Synchrotron Radiation Research Center. To enhance the resolution of infrared-image of single cells, synchrotron radiation rather than global was applied. In each cell line, spectra of 20 single cells were captured for further analysis.

### 3.5. Wax-Adsorption Kinetic Study with FT-IR Microspectrometry

Both paraffin and beeswax solutions were tested for their attachment onto cell membranes [27]. Before attaching wax solution to cells, the MirrIR low-e microscope slides must be washed by xylene for at least 10 min. Once the cells were fixed on MirrIR low-e microscope slides, the samples were briefly washed with xylene and subsequently dried with air. Infrared-images were collected at this step for comparison with further treatments. Subsequently, every sample was soaked in 5.5% of warm wax (paraffin or beeswax) solutions for two minutes, and then air-dried for further 10 min. After incubation with xylene again for 5 s to detach the residual wax from the cell membranes, infrared-images were collected. Similarly, additional soaking with xylene and FT-IR data collection were repeated to perform kinetics of wax-adsorption. In each sample, three single cells were monitored for their wax-adsorption kinetics.

### 3.6. Analysis of Spectra Data

We used the software OMNIC (v. 8.5; Thermo Fisher Scientific, Waltham, MA, USA) to capture chemical maps and spectra of each single ovarian cell. With the use of OMNIC, the functions of smooth and baseline correction were applied to each spectrum prior to the integration of IR absorbance of amide I and amide II. The level of wax attachment was computed by the software OPUS (v. 6.5, Bruker Optics, Ettlingen, Germany) and then displayed data in 3D maps. In order to integrate the absorption of lipid distributed on cells, we collected the data of lipid-region from three single cells for kinetic analysis.

## 4. Conclusions

In this work, we applied new methods to identify high methylation level globally in CP70 and demethylation by 5-aza. The methylation and 5-aza treatment conditions are correlated with paraffin-adsorption on cell membranes, and the paraffin-adsorption assay allows us to distinguish normal and cancer cell lines without labeling. These results implied significant alteration on membrane proteins caused by DNA methylation, and further studies on membrane proteins are essential. Overall, applying both chemical and physical detection methods on ovarian cancer cells provides us with a method of ovarian cancer detection and a future direction to elucidate the carcinogenic mechanism.
